# Water-Based Graphene Oxide–Silicon Hybrid Nanofluids—Experimental and Theoretical Approach

**DOI:** 10.3390/ijms23063056

**Published:** 2022-03-11

**Authors:** Gabriela Huminic, Alexandru Vărdaru, Angel Huminic, Claudiu Fleacă, Florian Dumitrache, Ion Morjan

**Affiliations:** 1 Mechanical Engineering Department 29, Transilvania University of Brasov, Bulevardul Eroilor, 500036 Brasov, Romania; gheorghe.vardaru@unitbv.ro (A.V.); angel.h@unitbv.ro (A.H.); 2 National Institute for Laser, Plasma and Radiation Physics, 409, Atomistilor Street, P.O. Box MG-36, Magurele, 077125 Bucharest, Romania; claudiufleaca@yahoo.com (C.F.); dumitracheflorian@yahoo.com (F.D.); ion.morjan@inflpr.ro (I.M.)

**Keywords:** graphene oxide sheets, silicon nanoparticles, hybrid nanofluid, viscosity, efficiency, correlation

## Abstract

In the current paper, a new hybrid nanofluid based on graphene oxide sheets and silicon nanoparticles is proposed for thermal applications. GO sheets and Si nanoparticles with different mixture ratios are dispersed in distilled water. Dynamic viscosity is measured at temperatures within the range 20–50 °C and the values are compared to the results available in the literature. The results indicated that the viscosity increases with increasing the mixture ratio of graphene oxide. A new correlation for the dynamic viscosity based on the experimental findings is proposed. Finally, the criteria for the performance of new hybrid nanofluid for thermal applications are analyzed.

## 1. Introduction

Graphene oxide is an attractive material due to its outstanding thermal, optical, and mechanical properties, and is used in various applications in engineering science. The main advantages of graphene oxide are a high thermal conductivity, requiring less pumping power, enhanced heat transfer ability, and being more stable [1].

The viscosity of the nanofluids is a relevant parameter in the study of heat transfer, because the pressure drop and implicitly pumping power depend on it. The main factors that influence this property are the viscosity of the base fluid, concentration of nanoparticles, morphology, and temperature. There are many studies on the thermo-physical properties of nanofluids in order to find an optimum ratio between the enhancement in thermal conductivity and the increase in viscosity, such that these nanofluids are deemed suitable for heat transfer applications. Recent articles on this topic have been published [1,2,3,4,5].

Hadadian et al. [6] investigated the thermal conductivity and viscosity of a graphene oxide/ethylene glycol nanofluid, and reported an enhancement in thermal conductivity of 30% at a concentration of 0.007% and 20 °C, while the viscosity increased 3.4-times for 0.005%.

A study on the thermo-physical properties of graphene oxide dispersed in the mixture of 40%:60% water and ethylene glycol, respectively, was performed by Ijam et al. [7]. The results indicated that viscosity of the studied nanofluid is increased up 35% at a temperature of 20 °C.

In another study, Kamatchi et al. [8] considered the thermo-physical properties of water/reduced graphene oxide nanofluid and found an increase in viscosity up to 12.32% for a concentration of 0.3 g/L.

Sedaghat and Yousef [9] performed a study on the thermal conductivity and viscosity of graphene quantum dot (GQD) nanoparticles dispersed in various base fluids (water, ethylene glycol, and water–ethylene glycol mixture (60%:40%)). The results revealed maximum increases in viscosity up to 119.30%, 152%, and 128% for water, ethylene glycol, and the mixture of water and ethylene glycol, respectively.

Mozaffarian et al. [10] studied graphene oxide dispersed in 1-butyl-3-methylimidazolium-bis(trifluoromethylsulfonyl)imide ([Bmim] [NTf2]) and found increases in the thermal conductivity and specific heat of 6.5% and 42%, respectively, for 2 wt% GO nanoparticles. In addition, they found an increase in heat transfer coefficient of 7.2% noticed for the 0.5 wt.% GO nanoparticles.

Cabaleiro et al. [11] studied the viscosity of graphene oxide/water nanofluids and found an increase in this property up to 130% for the studied concentrations (0.0005–0.1%).

The viscosity of graphene oxide/water nanofluids at temperatures within the range of 298–313 K and concentrations of 0.01 and 0.1 wt% was studied by Esfahani and Languri [12]. They found an increase in viscosity of 60% for 0.1 wt% GO and 298 K.

Ugwekar et al. [13] investigated the thermal conductivity, viscosity, and heat transfer and flow characteristics of a water-based GO-CuO nanocomposite. They found that the viscosity increased with increasing the concentration of the GO-CuO nanocomposite and decreased with increasing the temperature, which is a common trend of liquids. In addition, the results showed that the heat transfer coefficient increased with the increasing Reynolds number for different concentrations of the GO-CuO nanocomposite.

Mehrali et al. [14] carried out a study on the viscosity of reduced graphene oxide decorated with silver NPs (Ag-rGO) dispersed in deionized water. Measurements were performed in the range of 298–333 K for a concentration of 100 ppm, and the results indicated an increase in viscosity of 22% for rGO nanofluid and a lower relative viscosity increase for Ag-rGO nanofluids.

Thermo-physical properties of graphene oxide/Co_3_O_4_ hybrid nanofluids with various base fluids were studied by Sundar et al. [15]. For a 0.2 vol.% and 60 °C, the increase in thermal conductivity of water-based nanofluid and ethylene glycol-based nanofluid was 19.14% and 11.85%, respectively, while the increase in viscosity for both nanofluids was 1.70-times and 1.42-times for the same concentration and temperature.

Ranjbarzadeh et al. [16] carried out a study on the behavior of a water-based graphene oxide−silicon oxide nanofluid and their results showed a maximum increase in the viscosity of the hybrid nanofluid of 345% compared to the base fluid.

Afrand et al. [17] studied the graphene oxide dispersed in water flowing in a tube under air cross-flow. Different volume concentrations of water/graphene oxide nanofluid (0, 0.05, 0.1, and 0.2%) were investigated and the results indicated that the average Nusselt number and the friction factor increased up to 51.4% and 21%, respectively, compared to the base fluid.

The heat transfer and pressure drop of the water/graphene nanofluid in a two-tube heat exchanger under turbulent flow was investigated by Sadeghinezhad et al. [18]. They reported an increase in the heat transfer coefficient and heat transfer performance coefficient up to 160% and 1.77, respectively. In another study, Mehrali et al. [19] also studied the heat transfer characteristics of water/graphene nanofluid flowing through a horizontal tube, and found a thermal performance coefficient of 1.15.

Miao et al. [20] studied the heat transfer and mechanical friction reduction properties of graphene oxide/water nanofluids. An increase in the Nusselt number ratio from 1.02 to 1.18 was achieved with increasing the concentration of graphene oxide. For 0.1 vol.%, the friction coefficient decreased up to 71%.

The effect temperature and volume concentration on the thermal conductivity of graphene oxide−silicon carbine/water hybrid nanofluid was investigated by Karimipour et al. [21]. Their results showed an increase in the thermal conductivity of up to 33.2%, with an increase in both the volume concentration of the nanoparticles and temperature. In another study, Bach [22] experimentally and numerically investigated the thermal conductivity of graphene oxide/silicon dioxide hybrid nanofluids and found an enhancement in the thermal conductivity of approximately 27% for 1.0 vol.% 50 °C.

The selection of the appropriate nanoparticles and base fluid could enhance the overall efficiency of the thermal systems. In this study, water, graphene oxide sheets, and silicon nanoparticles were selected to investigate the physical properties and heat transfer characteristics. The choice of graphene oxide was based on a high thermal conductivity (~5000 W/(mK)) [23] and high surface area [24]. First, the graphene oxide sheets and silicon NPs were synthesized and characterized. In the next stage, the prepared suspensions with different mixture ratios between graphene oxide sheets and silicon NPs were investigated in terms of viscosity and efficiency at temperatures within the range of 20–50 °C.

## 2. Experimental Procedure

The improved Hummers method using Preformed Acidic Oxidizing Medium (PAOM) [25] was applied to synthesize the GO sheets. The Si NPs synthesized by the laser pyrolysis technique [26] were used for the preparation of GO-Si hybrid nanoparticles. The silicon nanoparticles (NPs) were synthesized in the absence of oxygen. After their exposure to ambient air and also by dispersing them in water in the presence of the ultrasonic horn, a silicon dioxide superficial layer [27] rich in Si-OH (silanol) groups formed. Graphene oxide is known to have a high concentration of hydroxy, carboxy, and epoxy oxygen-containing groups [28]. It is expected that those hydrophilic groups interact with water molecules, facilitating both GO and Si NP dispersion in this liquid medium. Moreover, the same groups can also intermediate the physical attachment of some Si NPs to suspended GO sheets via hydrogen bonds. The detailed experimental procedures concerning the synthesis of both the GO sheets and Si NPs have been detailed in a previous study [29].

The hybrid nanofluids were prepared by dispersing synthesized GO−Si hybrid nanoparticles in distilled water. In the first stage, the graphene oxide cream was homogeneously dispersed in 120 mL distilled water using a Hilscher UIP 1000 Homogenizator sonotrode together with a milk shaker roto-vibrating device for 30 min. An external cold-water bath with a copper coil with freshwater circulation inside was used for cooling the mixing vessel. In the next stage, the Si NP powder was introduced in a bottle together with the required amount of water in order to attain a total of 250 mL. The suspension was sealed, strongly shaken, and then poured in small portions under continuous sonotrode and rod mixing for 20–30 min over the corresponding GO dispersion. In order to reach hybrid aqueous nanofluids with a mixture ratio of 2 g/L Si NPs + 0.5 g/L GO and 1.5 g/L Si NPs + 1 g/L GO, respectively, different Si nanoparticle quantities were employed. After that, the mixing was continued for 1 h under external cooling. All hybrid nanofluids were prepared with a weight concentration of 0.25%.

**Figure 1:**
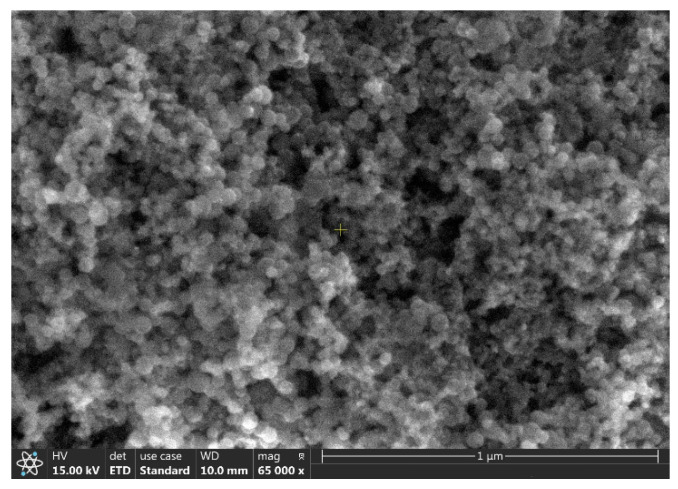
SEM image of Si nanoparticles from the corresponding Si nanofluids after drying.

**Figure 2:**
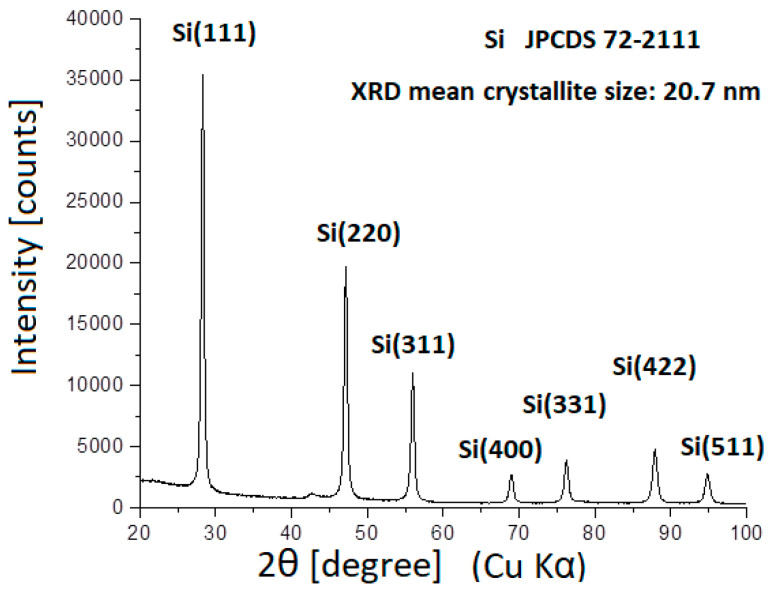
X-ray diffractogram of raw Si nanoparticle nanopowder.

**Figure 3:**
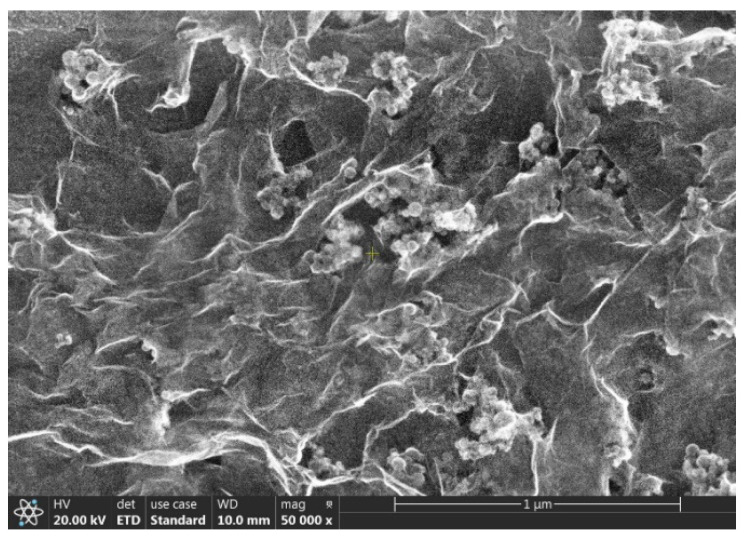
SEM image of the deposit containing Si NPs and GO nanofluid after 20× dilution with ethanol and drying.

Figure 1 shows the SEM image of the individual Si NPs. The diameter of the Si NPs varied between 15 and 50 nm and formed relative compact aggregates/agglomerates. The mean crystallite size calculated using the Debye−Scherrer formula was 20.7 nm (Figure 2). This value was compatible with the SEM picture, where the smaller particles seemed to be the majority. Figure 3 shows the SEM image of the hybrid nanofluid. The GO folded sheets seemed to cover or to surround clusters of one or several hundreds of nm in size composed of round Si aggregated NPs.

**Figure 4:**
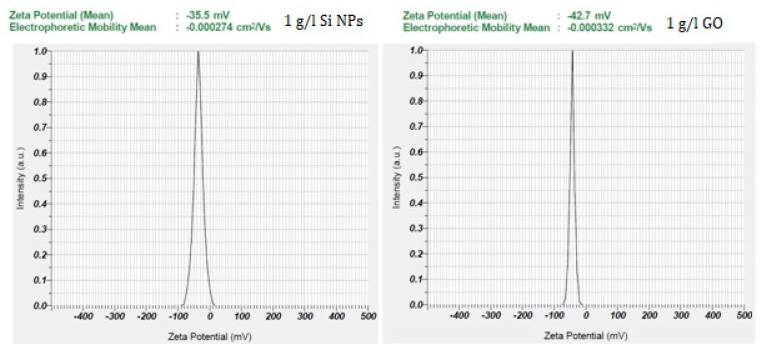
Zeta potential measurements.

For the evaluation of the stability of the studied nanofluids, the zeta potential measurements were performed on pure 1 g/L aqueous suspensions of GO and Si NPs. A good stability for both those nanostructures could be noticed due to their negative surface charging, which induced electrostatic repulsion between the particles (Figure 4).

The dynamic viscosity of the GO−Si/water hybrid nanofluid was measured with a Brookfield programmable viscometer with software for the complete external control of the viscometer. The viscometer was calibrated with the distilled water. The accuracy of the device was ±1% and its repeatability was ±0.2%. For the measurement of the viscosity at different temperatures, a thermostat bath, Haake C10-P5/U, with an accuracy of ±0.04 °C was used.

## 3. Results and Discussions

### 3.1. Dynamic Viscosity

**Figure 5:**
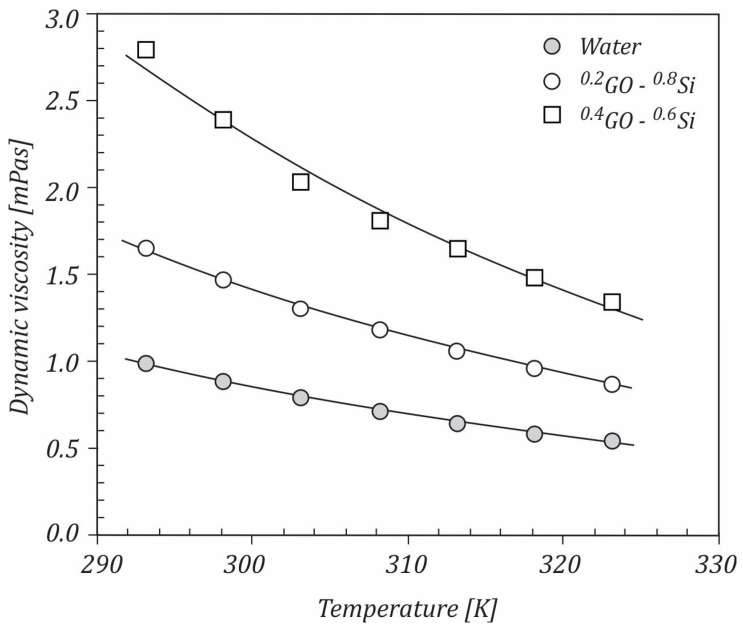
Variation of dynamic viscosity with temperature.

**Figure 6:**
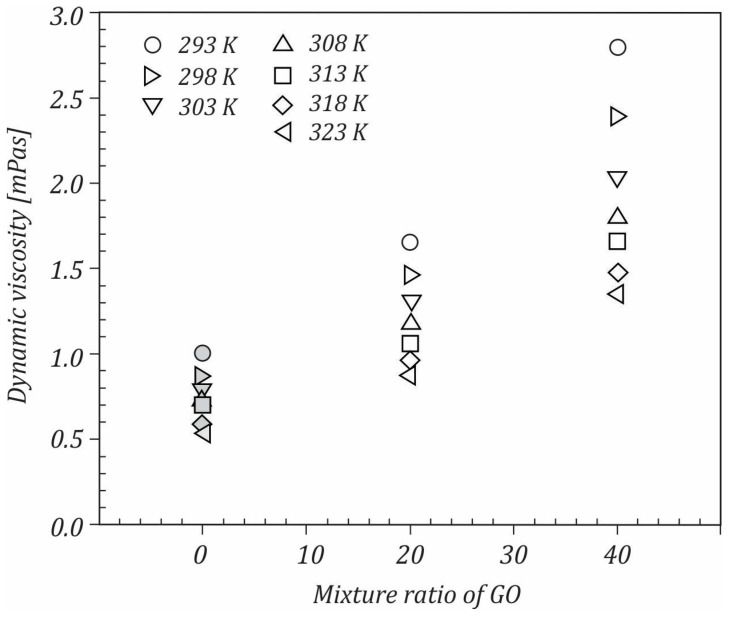
Variation of dynamic viscosity with the mixture ratio of GO sheets.

Figure 5 depicts the dynamic viscosity vs. temperature for both the water and 0.25 wt.% GO−Si hybrid nanofluid at various mixture ratios for GO sheets and Si NPs. According to the results, the viscosity exponential decreased with the increasing temperature, which is a general trend. As the temperature increased, the average speed of the molecules increased and thus the average intermolecular forces decreased. In addition, at a constant temperature, the dynamic viscosity increased as the content of GO sheets in the mixture increased. At lower temperatures, this increase was more obvious (Figure 6). The highest viscosity was noticed in the mixture ratio for GO sheets of 0.4 and the temperature of 293 K. One reason in the increase in viscosity may be the different shapes of the GO and Si components.

**Figure 7:**
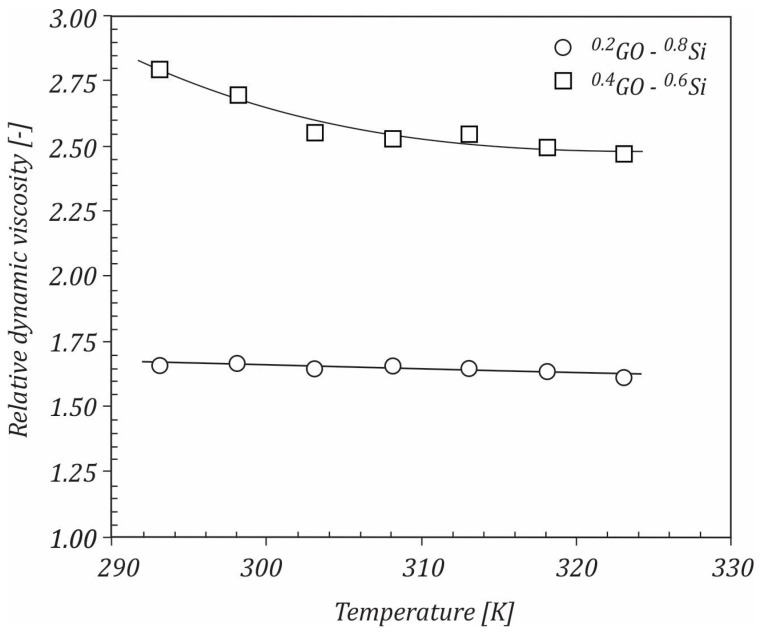
Variation of relative dynamic viscosity with temperature.

The variation of relative dynamic viscosity, defined as $\mu_{r}=\frac{\mu_{GO+Si/water}}{\mu_{water}}$, with the temperature at different mixture ratios of GO sheets, is shown in Figure 7. As can be seen, the viscosity ratio for ^0.2^GO-^0.8^Si hybrid nanofluid decreased slightly from 1.66 to 1.61 with increasing the temperature from 293 K to 323K compared to water, while for the ^0.4^GO-^0.6^Si hybrid nanofluid, the viscosity ratio varied in the range of 2.79–2.47 for the same temperature range.

**Figure 8:**
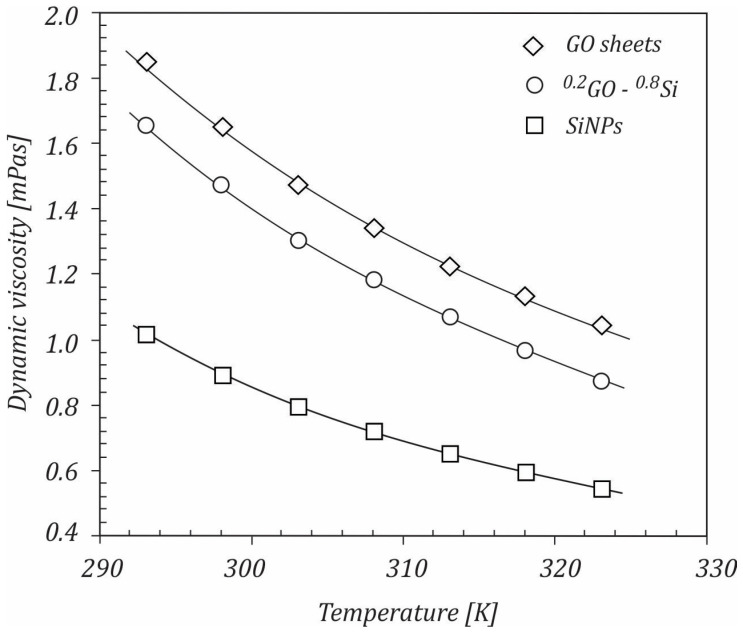
Comparison of the hybrid nanofluid with the water-based GO and Si nanofluids.

A comparison of the dynamic viscosity values achieved for the ^0.2^GO-^0.8^Si hybrid nanofluid with the results for mono nanofluids is depicted in Figure 8. It is seen that GO/water nanofluid had a higher dynamic viscosity compared to both the ^0.2^GO-^0.8^Si/water and Si/water nanofluids. Furthermore, it can be observed that adding GO into the Si nanofluid led to an increase in the dynamic viscosity of the hybrid nanofluid. It can be concluded that using the hybrid nanofluid could be a viable solution due to its lower viscosity compared to the GO nanofluid. GO sheets with a larger specific surface area interacted more strongly with the water molecules, thus leading to an increase in viscosity. By combining the GO sheets with Si NPs that had a smaller specific surface area, a lower viscosity for the hybrid nanofluid was achieved.

The dynamic viscosity values for mono and hybrid nanofluids were correlated by means of an exponential equation as a function of reduced temperature, defined as $T_{r}=\frac{T}{T_{0}}$:

(1) $$\mu =a\cdot e^{b\cdot T_{r}}\;[\mathrm{mPa}\cdot s]$$

where $T_{0}=273\;K$ and the coefficient values *a*, *b*, and $R^{2}$ are given in Table 1:

In the order comparing of the current results with the results reported in the open literature, the weight concentration is converted into the volume fraction using the following equation [30]:

(2) $$\phi =\frac{\varphi }{\rho_{p}\left(\frac{\varphi }{\rho_{p}}+\frac{1-\varphi }{\rho_{bf}}\right)}$$

where φ is the weight fraction, $\rho_{p}$ is the density of the particles (kg/m^3^), and $\rho_{bf}$ is the density of the base fluid (kg/m^3^). The physical properties of base fluid, GO sheets, and Si NPs at 25 °C are described in Table 2.

Table 3 defines the volume fractions for all of the studied mono-hybrid nanofluids.

**Figure 9:**
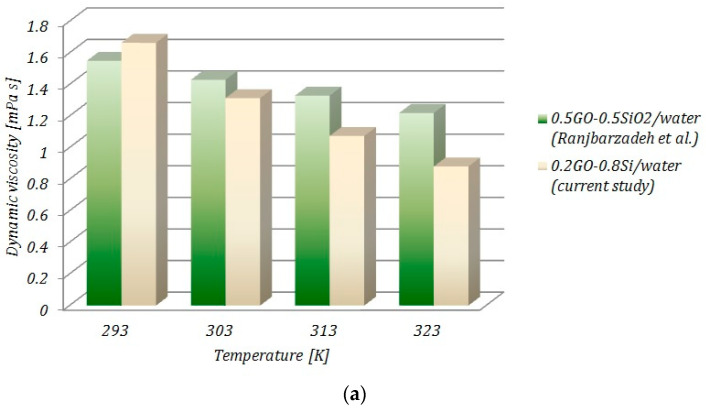

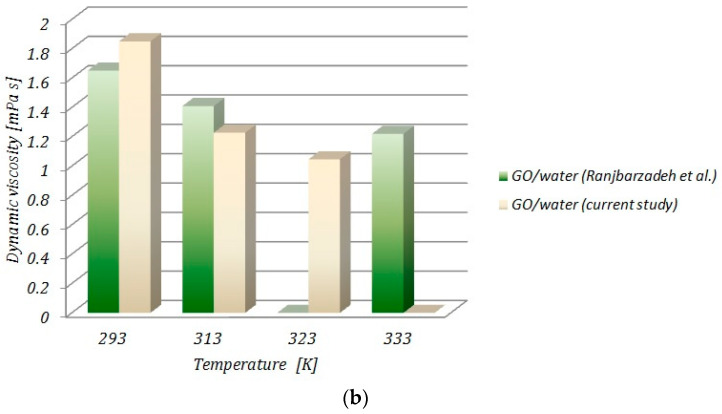
Comparison of the results for (**a**) hybrid nanofluid and (**b**) nanofluid with data from the literature.

Figure 9a,b draws a comparison with the data provided by Ranjbarzadeh et al. [16], who experimentally studied water-based graphene oxide−silicon oxide nanofluids. The results are plotted for a concentration of 0.1 vol.% GO+SiO_2_. As can be seen from Figure 9, the values of viscosity recorded in the current study were lower than the viscosities for GO+SiO_2_/water and graphene oxide/water, respectively, when increasing the temperature, excepting the temperature of 293 K. It can also be seen that a higher mixture ratio of graphene oxide led to an increase in the viscosity of the hybrid nanofluid.

### 3.2. Criteria of Evaluation of the Performance of the Hybrid Nanofluids Based on Graphene Oxide−Silicon

To compare the heat transfer performance of the studied nanofluids to their corresponding base fluids, the ratio of thermal conductivity was used as the figure of merit in the laminar flow [31]. For a turbulent flow, the following figure of merit described by the Vajjha and Das [32] is used:

(3) $$\frac{Mo_{hnf}}{Mo_{bf}}=\frac{\rho_{hnf}^{0.8}}{\rho_{bf}^{0.8}}\;\frac{c_{p,hnf}^{0.5}}{c_{p,bf}^{0.4}}\;\frac{k_{hnf}^{0.5}}{k_{bf}^{0.6}}\;\frac{\mu_{bf}^{0.4}}{\mu_{hnf}^{0.3}}$$

The density and specific heat for all nanofluids are calculated using Equations (4) and (5), while the dynamic viscosity and thermal conductivity are measured experimentally:

- density:
  (4) $$\rho_{hnf}=\phi \;\rho_{p}+\left(1-\phi \right)\rho_{bf}$$
- specific heat:
  (5) $$c_{p,hnf}=\phi \;c_{p,p}+\left(1-\phi \right)c_{p,bf}$$

**Figure 10:**
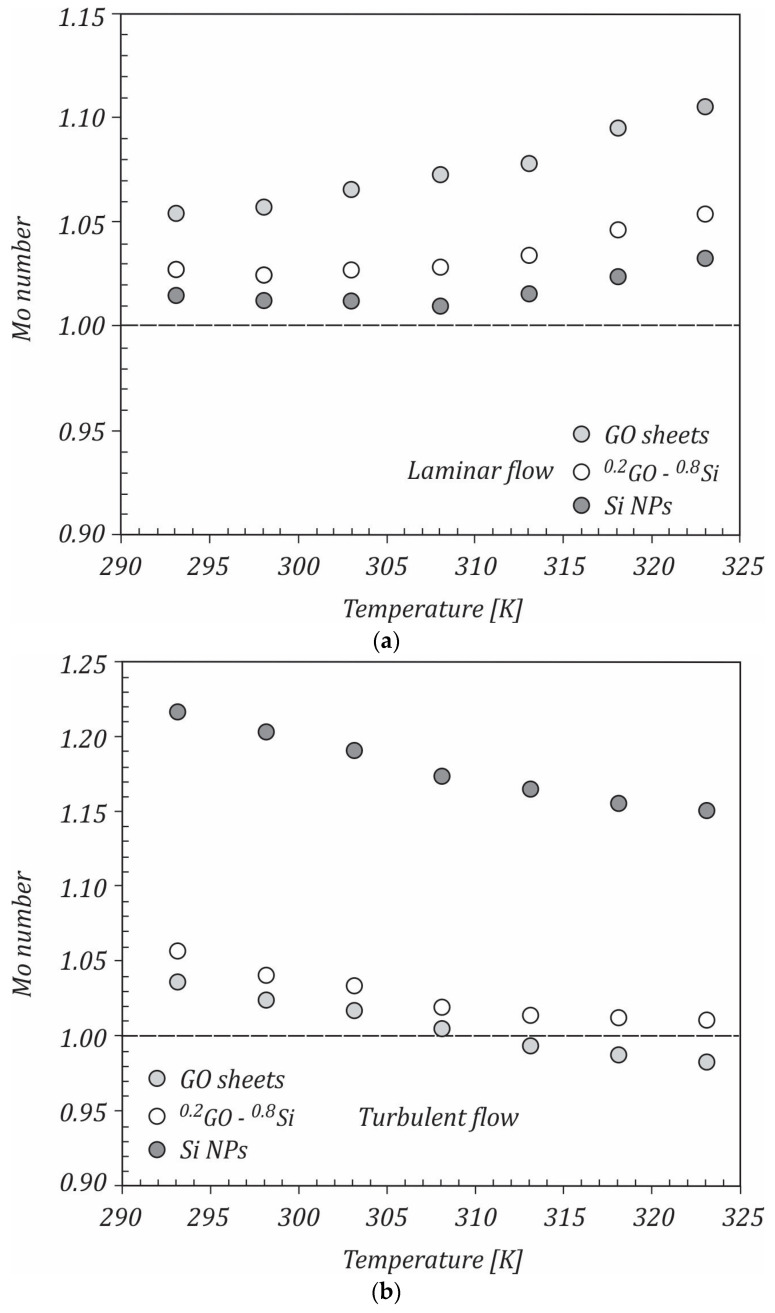
Mouromtseff number in: (**a**) the laminar flow and (**b**) the turbulent flow.

If the ratio of Mo Number (${\mathrm{Mo}}_{\mathrm{nf}}/{\mathrm{Mo}}_{\mathrm{bf}}$) is higher than 1, the nanofluid is considered as a good alternative to the base fluid. The computed ratios of the Mouromtseff number for the GO/water, ^0.2^GO-^0.8^Si/water, and Si/water nanofluids in the laminar and turbulent flows are shown in Figure 10a,b. It can be seen that in the laminar flow, all nanofluids improved the heat transfer performance over the base fluid, mainly due to the higher thermal conductivities of the nanofluids. With the increasing temperature, the thermal conductivities increased compared to the base fluid and the heat transfer performance of the nanofluids was better than that of the base fluid. In the turbulent flow, the opposite trend of the Mouromtseff number with the temperature was seen. The Mouromtseff number decreased with increasing the temperature, and at temperatures higher than 313 K, the Si/water nanofluid had values of the Mouromtseff number close to 1. It can be concluded that in the laminar flow, the studied nanofluids could be beneficial at high temperatures, while in the turbulent flow, the nanofluids were profitable at lower temperatures.

To determine the efficiency of the hybrid nanofluids in heat transfer applications under turbulent flow, the heat transfer coefficients ratio was analyzed. According to the Gnielinski correlation [33], the heat transfer coefficient ratio is given by the following [34]:

(6) $$\frac{h_{nf}}{h_{bf}}=\frac{Nu_{nf}}{Nu_{bf}}\frac{k_{nf}}{k_{bf}}$$

where the Nusselt number is:

(7) $$Nu=\frac{\left(\frac{f}{8}\right)\left(Re-1000\right)Pr}{1+12.5{\left(\frac{f}{8}\right)}^{0.5}\left(Pr^{2/3}-1\right)}$$

The Gnielinski Equation (7) is valid for 0.5≤Pr≤2000 and $3\times 10^{3}<Re<5\times 10^{6}$.

The Reynolds (*Re*) and Prandtl (*Pr*) numbers from Equation (7) are computed by taking into account the thermo-physical properties of studied nanofluids:

(8) $$Re=\frac{\rho wd}{\mu }$$

respectively

(9) $$Pr=\frac{\mu \;c_{p}}{k}$$

The friction factor f is determined by the first Petukhov equation:

(10) $$f={\left(0.790\cdot \ln Re-1.64\right)}^{-2}$$

**Figure 11:**
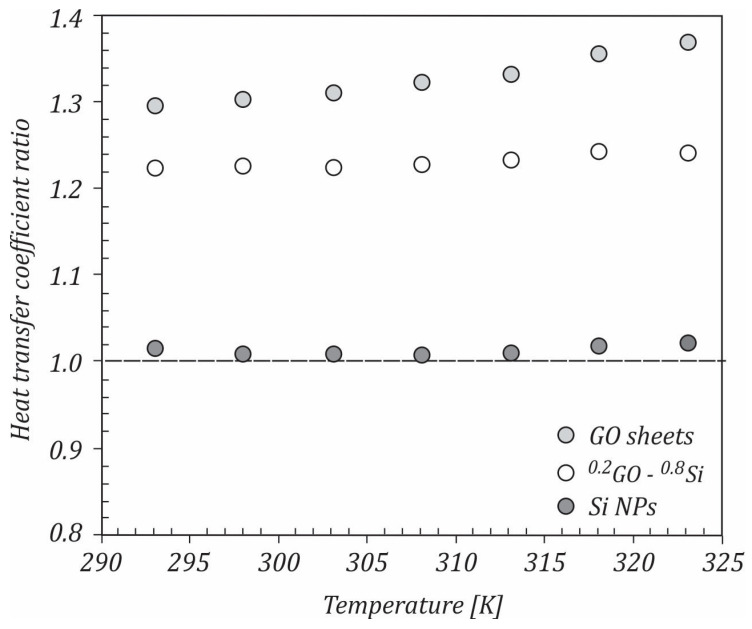
Heat transfer coefficients ratios.

The variation in the heat transfer coefficient ratio with temperature for the studied mono and hybrid nanofluids flowing at a Reynolds number of 10,000 is shown in Figure 11. If the ratio $h_{nf}/h_{bf}>1$, the efficiency of the system was considered to be improving. For all of the studied nanofluids, it could be seen that this ratio was higher than 1, with the maximum values being achieved for the GO sheet nanofluids. With increasing the temperature, the heat transfer ratio increased. At 323 K, the ratios of the heat transfer coefficients were 1.368, 1.244, and 1.022 for GO/water, ^20^GO-^80^Si/water, and Si/water nanofluids, respectively.

In some studies [34,35], the performance of the nanofluids was evaluated using the Dittus−Boelter correlation,

(11) $$Nu=0.023\cdot Re^{0.8}\cdot Pr^{n}$$

where n=0.4 for heating and 0.3 for the cooling of the fluid flowing through the tube. This equation is valid for Re>10000 and 0.7≤Pr≤160.

The Equation (11) may give errors as large as 25% [33]. By using the more complex, but accurate equations, such as the second Petukhov equation,

(12) $$Nu=\frac{\left(\frac{f}{8}\right)Re\cdot Pr}{1.07+12.5{\left(\frac{f}{8}\right)}^{0.5}\left(Pr^{2/3}-1\right)}$$

these errors can be reduced considerably to less than 10% [33]. The Petukhov correlation was valid for 0.5≤Pr≤2000 and $10^{4}<Re<5\times 10^{6}$.

Table 4 shows a comparative analysis of the results obtained for the ratios of the heat transfer coefficients using the Dittus−Boelter, Petukhov, and Gnielinski correlations at temperatures of 293 K and 323 K. As can be seen from Table 4, the Dittus−Boelter correlation gave values for the heat transfer coefficient ratio less of than 1, both for the GO/water and ^0.2^GO-^0.8^Si/water, while the Petukhov and Gnielinski correlations indicated values higher than 1 for all of the studied nanofluids.

Another way to evaluate the efficiency of hybrid nanofluids in heat transfer applications is

(13) $$\eta =\frac{h_{nf}/h_{bf}}{\Delta P_{nf}/\Delta P_{bf}}$$

where the pressure drop ratio is computed using the following relation:

(14) $$\frac{\Delta P_{nf}}{\Delta P_{bf}}=\frac{f_{nf}}{f_{bf}}\frac{\rho_{nf}}{\rho_{bf}}$$

**Figure 12:**
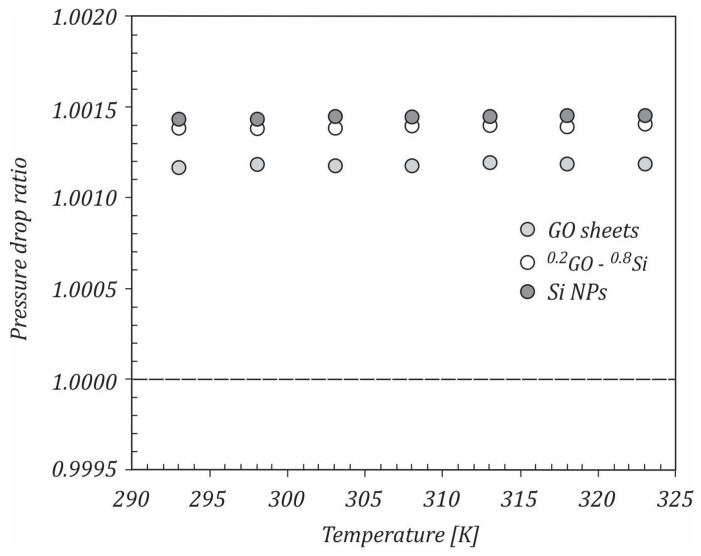
The variation of the pressure drop ratio with temperature.

**Figure 13:**
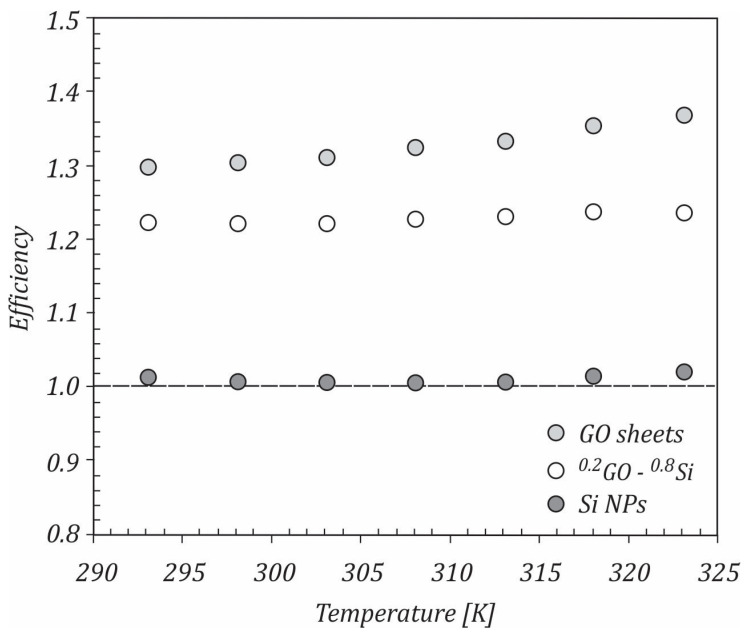
Efficiency of the studied nanofluid.

In this case, if η>1, the nanofluid can be useful for heat transfer applications. The variation of the pressure drop ratio with temperature is illustrated in Figure 12. As shown, the pressure drop ratio was almost constant with the increasing temperature for all nanofluids, which means that the temperature did not have a direct effect on the pressure drop. The values of the pressure drop ratio were close to 1, with the maximum values being achieved for the GO/water nanofluid, followed by the ^0.2^GO-^0.8^Si/water and Si/water nanofluids. Using the heat transfer coefficient and pressure drop ratios, the efficiency was calculated and is depicted in Figure 13.

Heat transfer enhancement in heat transfer applications is usually accompanied by an increased pressure drop, and, implicitly, by a higher pumping power. Therefore, any gain in heat transfer enhancement should also take into account the increases in the pumping power. The pumping power [36,37] for the turbulent flow was evaluated based on the dynamic viscosity and density of the considered fluids:

(15) $$\frac{W_{hnf}}{W_{bf}}={\left(\frac{\mu_{hnf}}{\mu_{bf}}\right)}^{0.25}\;{\left(\frac{\rho_{bf}}{\rho_{hnf}}\right)}^{2}$$

**Figure 14:**
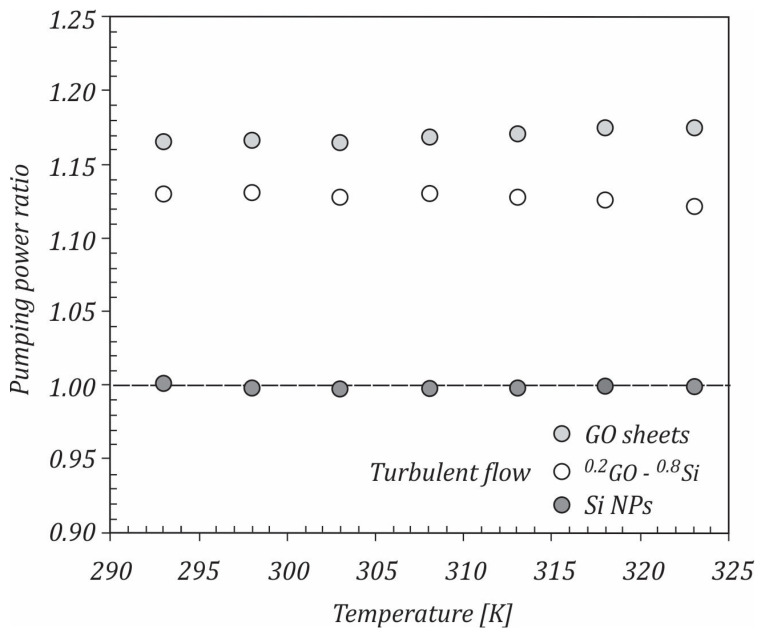
Pumping power variation with the temperature.

If the ratio is $\frac{W_{hnf}}{W_{hnf}}<1$, the nanofluids can be considered beneficial for heat transfer applications. The calculated pumping power ratios for the studied nanofluids are shown in Figure 14. It can be seen that Si/water nanofluid had pumping power ratios close to 1, while GO/water and ^0.2^GO-^0.8^Si/water nanofluids had pumping power ratios higher than 1 for all of the temperatures, mainly due to the higher dynamic viscosities of the nanofluids.

## 4. Conclusions

In the current paper, a new hybrid nanofluid was investigated. Firstly, the silicon NPs and graphene oxide sheets were synthesized using the laser pyrolysis technique and improved Hummer method, respectively, and were characterized using SEM and X-ray diffraction techniques. In the second stage, the dynamic viscosity values of water-based GO−Si hybrid nanofluids were measured. A new correlation for the dynamic viscosity based on the experimental findings was proposed. The results achieved from experiments are as follows:

- The increase in the graphene oxide content led to dynamic viscosity rising.
- The current results were compared with the research from the available literature.
- In laminar and turbulent flows, the studied nanofluids showed an improvement in efficiency.
- Heat transfer coefficient ratios in the turbulent flow for all of the studied nanofluids were higher than 1, with the maximum values being achieved for the GO sheet nanofluids.
- The pumping power ratio for Si/water nanofluid was close to 1, while for GO/water and ^0.2^GO-^0.8^Si/water nanofluids, the pumping power ratios were higher than 1 for all temperatures.
- A correlation for dynamic viscosity was proposed in this study. The equation is valid for temperature in the range of 25 °C to 50 °C.

## Figures and Tables

**Table 1:** Coefficient values *a*, *b*, and $R^{2}$ obtained by fitting Equation (1).

Nanofluid	*a*	*b*	$R^{2}$
GO sheets	455.33	−5.15	0.9939
Si NPs	386.33	−5.554	0.9945
^20^GO-^80^Si	787.72	−5.75	0.9986
^40^G0-^60^Si	2952.9	−6.523	0.9874

**Table 2:** The physical properties of base fluid, GO sheets, and Si NPs at 25 °C.

Nanoparticles/Base Fluid	$\mathrm{Density}\;[\mathrm{kg}/m^{3}]$	Specific Heat [J/kgK]
Graphene oxide sheets (GO) [15]	1910	710
Silicon (Si)	2329	700
Water	997	4181.6

**Table 3:** The volume fractions of the suspensions.

Nanofluid	Weight Concentration [%]	Volume Fraction [-]
GO sheets	0.25	0.001307
Si NPs		0.001112
^20^GO-^80^Si		0.001073

**Table 4:** Heat transfer coefficient ratio.

Nanofluid	GO/Water		^0.2^GO-^0.8^Si/Water		Si/Water	
Temp.	293 K	323 K	293 K	323 K	293 K	323 K
Dittus-Boelter correlation	0.808	0.819	0.83	0.853	1.002	1.017
Petukhov correlation	1.306	1.381	1.235	1.253	1.016	1.021
Gnielinski correlation	1.297	1.386	1.226	1.242	1.016	1.022

## Data Availability

Not applicable.
